# Coproducing COVID‐19 Health Information Resources: A Participatory Study With Older Adults From Minoritised Ethnic Communities in the UK

**DOI:** 10.1111/hex.70370

**Published:** 2025-08-07

**Authors:** Priyamvada Paudyal, Aghna Wasim, Saliha Majeed‐Hajaj, Naresh Khapangi Magar, Rebecca Sharp, Emily Skinner, Arya Sharma, Laura Hughes, Debbie Isobel Keeling, Jo Armes, Kavian Kulasabanathan, Krysia Canvin, Santosh Gaihre, Jackie Cassell

**Affiliations:** ^1^ Institute for Global Health and Wellbeing, School of Medicine Keele University Staffordshire UK; ^2^ Department of Primary Care and Public Health Brighton and Sussex Medical School Brighton UK; ^3^ Strategy and Design Camden Council London UK; ^4^ Centre for Nepal Studies London UK; ^5^ Health Innovation Kent Surrey Sussex Crawley UK; ^6^ Peterhouse University of Cambridge Cambridge UK; ^7^ King's College London ‐ Strand Campus London UK; ^8^ University of Sussex Business School University of Sussex Brighton UK; ^9^ School of Health Sciences University of Surrey Guildford UK; ^10^ Imperial College Healthcare NHS Trust London UK; ^11^ Institute of Applied Health Sciences, School of Medicine, Medical Sciences and Nutrition University of Aberdeen Aberdeen UK; ^12^ Centre for Health Service Studies University of Kent Kent UK

**Keywords:** coproduction, COVID‐19, leaflets, minoritised ethnic communities, older adults, participatory study

## Abstract

**Background:**

Minoritised ethnic communities in the UK experience disproportionate levels of morbidity and mortality compared to their Caucasian counterparts. This disparity was magnified during the COVID‐19 crisis, particularly amongst older adults. An effective way to target such inequalities is through health communication, but language barriers and cultural differences can make this challenging. This study was conducted during the midst of the COVID‐19 pandemic and aimed to coproduce culturally, linguistically, and age‐appropriate COVID‐19 health education resources tailored to the needs of older adults from communities facing such challenges.

**Methods:**

This multi‐method participatory study was focused on the information needs of older adults (65+ years) from Nepalese and Indian communities in Southeast England. The study consisted of three interconnected phases: 1) a qualitative study using semi‐structured interviews and an informal literature review; 2) coproduction of COVID‐19 resources using participatory workshops; and 3) dissemination of the resources.

**Results:**

We interviewed 24 participants: 13 older adults, seven family members and four healthcare providers. Findings revealed varying level of COVID‐19 knowledge with language and illiteracy cited as key barriers to accessing health information. Participants highlighted the importance of culturally sensitive messages and appropriate means of dissemination, such as community centres and places of worship. Drawing on these findings, culturally and age‐appropriate COVID‐19 information leaflets were coproduced in Hindi and Nepalese through participatory workshops and underwent subsequent iterative refinement. Digital and printed versions of the final copies were then distributed to communities and stakeholders.

**Conclusion:**

We adopted an inclusive and participatory approach to formulating culturally relevant information resources on COVID‐19. The coproduction process, findings, and reflections from this study may be useful in informing future public health programmes and policies targeting other underserved groups.

**Patient and Public Contribution:**

Two community members were actively involved at every stage of the study. They contributed to the refinement of the interview guide, discussion on the key findings, and dissemination of coproduced resources.

## 1 Introduction

Since its emergence in December 2019, COVID‐19 has infected around 777 million people and claimed over 7.1 million lives globally [1, 2]. As of 4 August 2024, some of the highest rates of COVID‐19 morbidity and mortality have been noted in the UK, with over 25 million cases and 232,000 deaths attributed to the disease [3, 4]. A disproportionate degree of this mortality and morbidity burden fell on minoritised ethnic communities. Individuals in the UK with Black, Middle Eastern, and South Asian backgrounds were reported to be at increased risk of contracting COVID‐19 compared to their White counterparts [5]. Several reviews also highlighted an increased likelihood of adverse outcomes, including hospitalisation, critical care admission, need for invasive treatment, and mortality among minoritised ethnic communities [6, 7, 8, 9].

The increased risk and severity of COVID‐19 infection seen in older adults was reported to be compounded by the presence of age‐related vulnerabilities which exacerbate susceptibility to infection and subsequent deterioration of health [10, 11, 12, 13]. The age‐related effects of COVID‐19 were attributed to co‐morbid chronic health conditions e.g., cardiovascular disease, diabetes, hypertension, and kidney disease, which have a higher prevalence in minoritised ethnic populations compared to White individuals [14, 15]. Members of these communities are also more likely to be employed as front‐line professionals in insecure jobs (e.g., zero hour contracts), often coupled with generally high levels of deprivation and low savings, particularly for those over 55 [16]. This economic disadvantage often impacts their access to safe housing, leading to overcrowding and multi‐generational living [17]. Our previous qualitative study found that multigenerational living offers some benefits such as younger family members supporting daily activities, helping understand health information, and navigating the healthcare system, while older adults often provide childcare support [18]. However, these living arrangements also makes it difficult for cohabitants to follow social distancing, self‐isolation and shielding, facilitating higher rates of COVID‐19 transmission within the household [17]. Furthermore, lack of trust in the healthcare system and structural racism have been reported to contribute to further marginalisation, worsening the health outcomes in this group [19].

Reducing the risk of COVID‐19 and improving health outcomes required effective communication of relevant health information. In older adults from minoritised ethnic communities, tailored approaches addressing language barriers and cultural differences were key to enable positive engagement and understanding [20, 21, 22]. This illustrates a need for culturally appropriate health interventions that use cross‐cultural messaging such as cultural references or visuals and translated informative material. Culturally sensitive interventions have been found to be better received by minoritised ethnic communities, and in turn, more effective in increasing the uptake of positive health behaviours [23, 24]. Public health guidance around COVID‐19 had been largely delivered in English without consideration for cultural nuances, suggesting that appropriate health messages may not have reached to these communities who are disproportionately affected by the disease. Providing accessible and understandable information was crucial to educating, empowering, and improving COVID‐19 related health outcomes among minoritised ethnic communities.

Culturally informed interventions and resources can be developed through collaborative approaches such as community‐based participatory research and coproduction activities. These activities are based on equitable partnerships between researchers and community members, with the aim of combining their knowledge and collective problem‐solving for social issues [25]. Coproduction is grounded in principles of inclusivity, reciprocity, and shared responsibility and power [26, 27, 28]. It is particularly important for ensuring that research outputs meet the needs of vulnerable populations, such as minoritised ethnic communities who remain underrepresented when it comes to research participation and public engagement [29, 30]. Lack of engagement in research may stem from historical injustices and socioeconomic inequalities culminating in feelings of distrust and wariness of authorities, institutions, and the state more generally [31]. Coproduction approaches involve seeking input and involvement of marginalised groups in the planning, development, execution, and dissemination of research targeted towards themselves, signifying the value of their individual and collective experiences [31]. This may in turn promote a sense of ownership, belonging, and empowerment among these populations, potentially addressing historical distrust and increasing their willingness to partake in research [31]. Although coproduction strategies have shown promise in reducing health inequalities [32, 33], they remained under‐utilised in the context of alleviating the COVID‐19 burden in minoritised ethnic communities through culturally informed health education. This study, therefore, aimed to codesign and coproduce culturally, linguistically, and age‐appropriate health education resources on COVID‐19, tailored to the needs of older adults from Indian and Nepalese backgrounds.

## 2 Materials and Methods

This multi‐method participatory study was conducted during the midst of the COVID‐19 pandemic (July‐December 2020). During the planning of this study, inividuals from Asian communities had the highest burden of critical illness from COVID‐19 (14%) among minoritised ethnic communities in the UK [34]. In particular, older adults from South Asian communities were especially vulnerable to COVID‐19, due to greater likelihood of sharing living spaces with younger people, and possessing more co‐morbidities such as hypertension, diabetes, and cardiovascular disease [35]. Therefore, we focused on the informational needs of older adults (65+ years) from these communities in Kent, Surrey, and Sussex.

**Figure 1:**
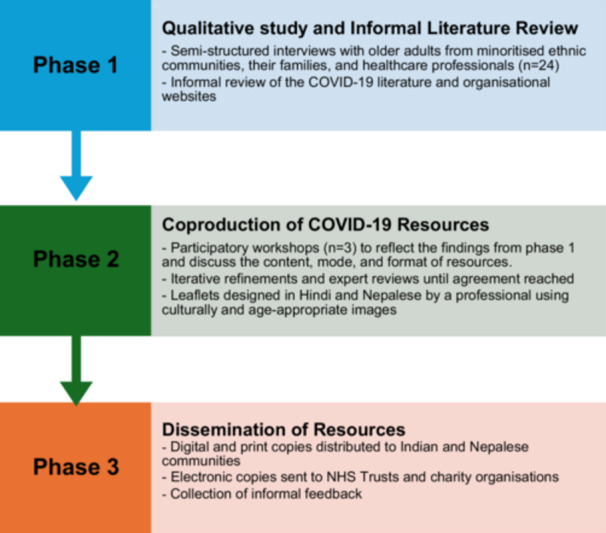
Study flow diagram.

The UK Government guidelines on social distancing were adhered to throughout the study and virtual techniques on qualitative data collection, feedback, and iterative coproduction processes were integrated in the design. The study was funded under rapid response to the COVID‐19 situation. Despite the urgency, it was crucial to dedicate time to build meaningful relationships with communities to ensure their involvement as full co‐partners, explore their current understanding and beliefs regarding COVID‐19, and identify knowledge gaps. Additionally, it was important to determine what information should be included and the most effective format for delivery of the information. As such, this study consisted of three interconnected phases incorporating these aspects (Figure 1).

### 2.1 Phase 1: Qualitative Study to Identify the Information Needs of Older Adults

We conducted a qualitative study using semi‐structured interviews to explore key information needs of older adults from Indian and Nepalese backgrounds (aged 65 and over), their relatives (aged 18 and over), as well as healthcare professionals (aged 18 and over) who engaged with these communities during the COVID‐19 pandemic. Researchers networked with community organisations, places of worship, and local authorities to identify potential participants. Purposive and snowball sampling were used to recruit eligible members of Indian or Nepalese communities with the help of leaders from respective communities. Verbal informed consent via the telephone was sought before the interview and recorded using a digital voice recorder. The interviews explored older adults and their family members' experience of lockdown; their knowledge and information sources on COVID‐19;, preventative measures undertaken and health services uses and their perception on the health information they required further. Information was gathered on healthcare professionals' experiences of working with older Indian or Nepalese communities and their engagement with healthcare services during the pandemic and the support needs. All interviews were conducted by telephone (except one by Zoom) and participants had the option of being interviewed in Nepalese (by NK), Hindi (by SMH), or English (by SMH or ES). The interviews in Nepalese were translated professionally into English and transcribed verbatim. Interviews conducted in Hindi were translated and transcribed by the researcher, and interviews conducted in English were professionally transcribed. The data was analysed in NVivo (V.10) using thematic content analysis. Further details of the qualitative study including recruitment challenges, key themes and subthemes and the limitations of the study have been published elsewhere [18].

In addition to the qualitative interviews, we undertook a rapid unstructured literature review of the latest and emerging public health evidence at the time to ensure that the resources we produced were evidence‐based, current, and in accordance with the UK Government's advice and guidelines. Key words related to COVID‐19, minoritised ethnic communities, and health communication and information were used to capture literature published until August 2020 in EMBASE and Ovid MEDLINE. Furthermore, websites of the various organisations such as NHS trusts, the UK government, local authorities, Public Health England, community and charity organisations (e.g., South Asian Health Foundation, Race equality Foundation), academic journals (BMJ Open, BMC Medicine) and international organisations such as UNHCR and IOM were reviewed to explore the already existing resources for these communities and identify gaps for further resources in this area.

### 2.2 Phase 2: Coproduction of COVID‐19 Resources

Two coproduction steering groups were formed, one for the Nepalese communities and one for the Indian communities (*n* = 6 members per group), consisting of older adults from the communities and their family members who participated in phase one, and additional community volunteers and researchers. We ensured that there was good representation of participants with a range of different characteristics fromthese communities, including age, sex, and religion. Once the coproduction team was formed, informal meetings and telephone calls (up to three calls) were held to allow sufficient time to build mutual trust and encourage active involvement with the group members, by reducing psychosocial barriers [36]. Before the coproduction meeting, the groups were provided with information about the coproduction process and the value of their engagement, experience, and input in shaping the outcomes. This included information on their expected contributions to the process, the importance of respecting diverse perspectives and offering constructive feedback, and fostering a non‐judgmental environment. Additionally, the anonymity and confidentiality of the discussions were emphasised, as well as the voluntary nature of participation.

Once the preliminary findings from phase one was available, we held two coproduction workshops via zoom with theIndian steering group (facilitated by SMH and PP) and one with the Nepalese steering group (facilitated by PP and NKP). The workshops lasted approximately two hours each and were focused on reflecting upon the findings from phase 1, the literature review and the exploration of websites containing COVID‐19 information resources targeted at the minoritised ethnic communities at the time. The workshops encouraged active participation and collaboration, with each member ensuring that the voices of older adults and families were heard whilst actively involving them in the codesign process. Drawing on the findings from phase 1 and the emerging COVID‐19 literature at the time, the format and content of the educational resources and the most appropriate media for information delivery were discussed. During these discussions, the groups suggested that leaflets or booklets would be the preferred format for COVID‐19 educational resources, in addition to video resources with healthcare professionals of similar ethnic background, and radio or television messages. Participants highlighted that printed, folding leaflets or booklets with culturally relevant images, including key facts regarding symptoms, prevention, and testing along with key contact numbers would be useful for a quick assessment in times of need. The prioritisation to coproduce leaflets instead of other formats was made upon the communities' preference for the digital and printed leaflets, and the feasibility of realistically coproducing resources within the six months study duration.

The research team continued to work with the coproduction group after the workshops via informal meetings, emails, or telephone calls to draft the leaflets. Once the drafts were prepared in Hindi and Nepalese through iterative processing and group approval was secured, they were then reviewed by public health experts in the team (PP, JC, KK). This was during a period of rapidly evolving advice and information, as scientists gained new insights into transmission and infection, and before any vaccination programme had been established. Therefore, it was crucial to ensure the leaflets' content remained relevant and evidence based. A professional designer was then invited to design the leaflets using culturally relevant images. The study team worked closely with the designer to ensure that the images and information were clear and represented the cultural context of the communities. Special attention was given to the representation of older adults, visual appeal, cultural contexts, and accessibility of the resources, as well as government restrictions and advice at the time. The images used in the leaflets, including cultural symbols, attires, and lifestyles, were carefully selected through iterative discussions with the coproduction team, key researchers PP and NKM, who have Nepalese backgrounds, and SMH, PP, and NKM, who are familiar with Indian cultural contexts. Additionally, the leaflet designer, who has a strong connection to and understanding of both Indian and Nepalese cultures, contributed to the selection process. Emphasis was given to ensure that the visual in the leaflets genuinely resonated with the cultural contexts of the communities and did not rely on general assumptions. To further ensure the relevance, meaningfulness and appropriateness, the leaflet drafts were circulated to six additional Nepalese and Indian community members (three Indian and three Nepalese) outside the study team for feedback. Community members were compensated for their time and involvement in the coproduction through supermarket vouchers. The final resources were reviewed by the coproduction group before dissemination.

### 2.3 Phase 3: Dissemination of the Coproduced Resources

Throughout the project duration, we collaborated closely with family members, faith leaders, and community volunteers from partner organisations to help disseminate the coproduced leaflets among older adult communities. The leaflets were distributed to practitioners, providers, and commissioners across health and care as well as diversity and inclusion leads. They were also handed out to clinical organisations, academic organisations, local and regional councils, community groups, the voluntary sector such as minoritised ethnic communities organisations, faith organisations, and through social media channels. Moreover, the leaflets were shared through the NIHR Applied Research Collaboration and Health Innovation Network (formally the Academic Health Science Network) to encourage national dissemination. A Community of Practice event that included community groups, public health colleagues, and health and care practitioners across Kent, Surrey, and Sussex was organised to share the coproduction process and launch the coproduced resources.

We were unable to formally assess the impact of the leaflets due to time and resource constraints. However, we gathered some informal feedback on whether they helped to improve individuals' understanding of health information related to COVID‐19, increased their knowledge of taking preventative measures, improved communication with healthcare professionals, and facilitated the effective use of health services, including those unrelated to COVID‐19.

### 2.4 Patient and Public Involvement

Two community members were involved in the overall study design from the start. These members contributed to the refinement of the interview guide (advised on comprehensiveness of the interview guide and clarity of the language) and discussion on the relevance and importance of the key findings from the study.

## 3 Results

### 3.1 Phase 1: Findings From the Qualitative Study

A total of 24 participants; 13 older adults (aged 65 and over) from minoritised ethnic communities, seven family members, and four healthcare professionals were included. Thematic analysis of the data identified four themes: (1) COVID‐19 awareness, understanding and health information needs, (2) the living, social and community context in which people receive, discuss and action their health information, (3) interaction with healthcare, and barriers and facilitators for receiving health information and (4) the impact of COVID‐19 on health information needs and self‐care strategies.

The study revealed varying levels of COVID‐19‐related knowledge, ranging from comprehensive to limited, with many participants acknowledging the importance of ongoing awareness efforts.

Risk perception in older adults was often guided by their sense of identity; for example, the belief that being from Nepalese or Gurkha communities made them less prone to the virus and its effects, as noted by one healthcare professionals. Older adults typically resided in a multi‐generational setting with their families. Many older adults valued offers of support extended to them by members of their community and religious groups early in the pandemic. Whilst some participants were happy with the quality of healthcare they received, others expressed dissatisfaction. Several participants reported using traditional, complementary, or alternative therapies for promoting general wellbeing, despite only a few individuals believing that these would be effective management for COVID‐19. Participants reported obtaining information about the pandemic from email, newspapers, radio, television, government websites, academic journals, and social media. Cultural understanding within the healthcare setting was frequently brought up by participants. They emphasised the need for information in their own languages and encouraged culturally sensitive means of dissemination (e.g., community centres and places of worship). Details of this qualitative study have been published elsewhere [18].

### 3.2 Phase 2: Coproduction of Information Leaflets

The coproduction workshops lasted approximately two hours each. Findings from phase 1 were shared by the researchers in the first workshop. Participants then discussed the findings, shared their experience of living in the context of COVID‐19, and the importance of having useful, culturally relevant and reliable information resources during the rapidly evolving pandemic. The second workshop held with Indian communities focused on the nature and content of health education resources, preferred format and modes of delivery, and dissemination ideas. It was not possible to organise the second coproduction workshop with the Nepalese communities due to logistical reasons and unavailability of the members of the Nepalese coproduction committee. The aforementioned items were thus discussed with them via informal meetings and telephone calls.

**Figure 2:**
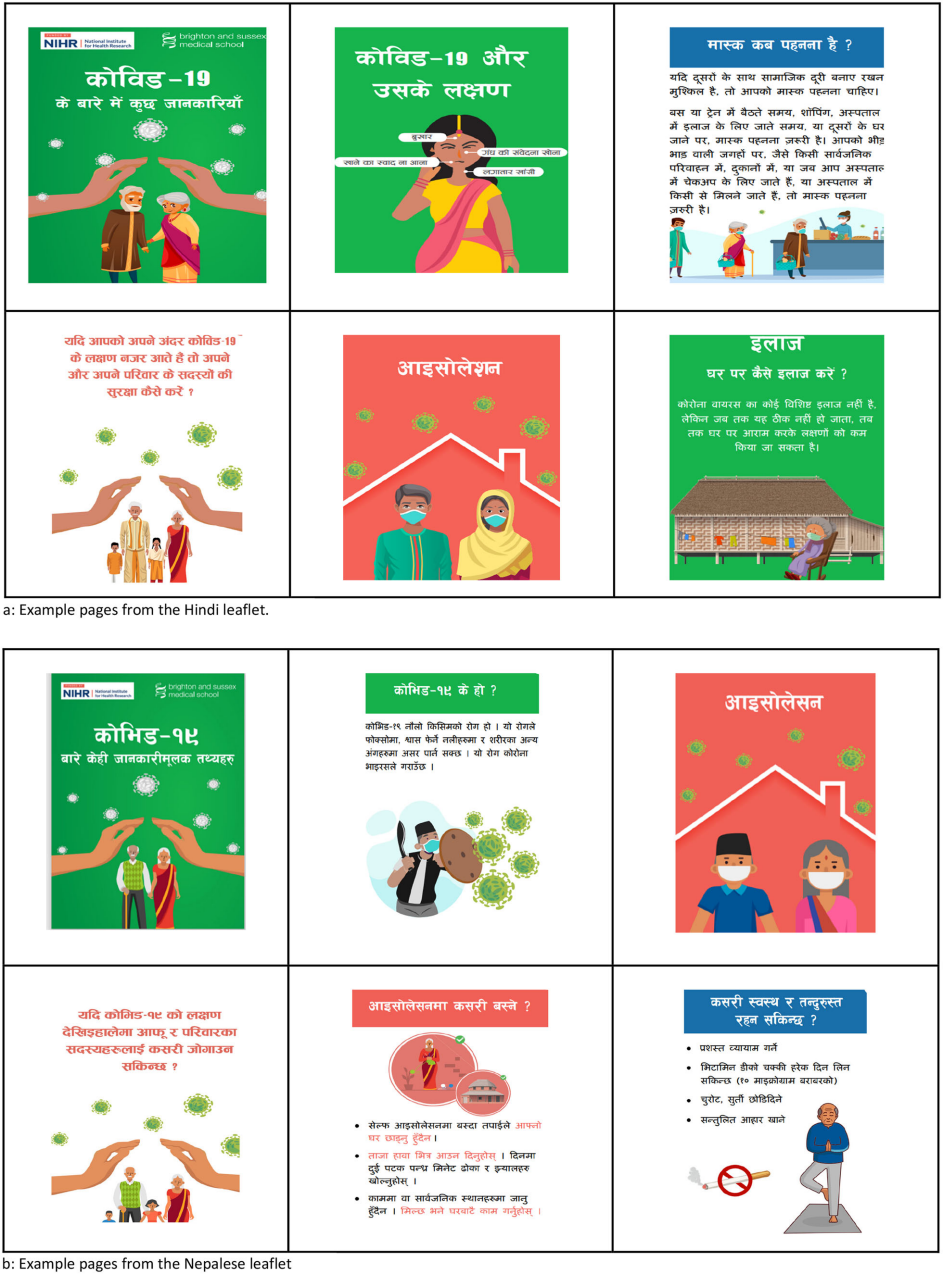
(a) Example pages from the Hindi leaflet. (b) Example pages from the Nepalese leaflet.

Based on the decision by the coproduction groups and considering the study's resource constraints, digital and printed leaflets providing clear and relevant public health information about COVID‐19 were coproduced for Hindi‐ and Nepalese‐speaking communities. The development of the leaflets consisted of numerous iterative phases and continual refinement until agreement was reached between all members of the coproduction group. The final leaflets contained the following information: (1) what is COVID‐19; (2) what are its main symptoms; (3) how it spreads; (4) protective and preventative measures; (5) self‐isolation guidance (i.e., when and how to self‐isolate); (6) treatment at home; (7) contacting health services; (8) COVID‐19 testing; and (9) a link to additional medical guidance from the NHS. The 28‐page foldable leaflets in Hindi and Nepalese can be accessed from the project website Co‐REM ‐ BSMS. Figure 2a,b represent example pages from the leaflets. The leaflets featured culturally relevant and age‐appropriate avatars dressed in vibrant, richly coloured traditional Indian and Nepalese attire. They also incorporated intricate details that reflect the lifestyle and traditions of these communities, such as yoga, knitting, and intergenerational living arrangements. Additionally, symbols like the Khukuri (a Nepalese knife representing power, strength, and loyalty) and other cultural elements were thoughtfully integrated while designing the leaflets.

### 3.3 Phase 3: Dissemination of the Coproduced Resources

We disseminated 2000 printed copies each of the Nepalese and Hindi leaflets across Kent, Surrey, and Sussex communities directly via post. Digital leaflets were also disseminated via emails, WhatsApp and social media to practitioners, providers, commissioners, public health academics, and researchers in the region. Moreover, the coproduced resources were picked up by international media outlets and audiences, with Nepalese media translating the resources into local dialects for dissemination via 20FM radio stations at a time whilst the crisis was unfolding in Nepal and COVID‐19 infection rates were soaring. The innovative nature of the leaflets was praised by Nepalese media portals in both the UK (wenepali.com) and Nepal (Kathmandu post). In addition, the leaflets were widely shared by community members on social media platforms including Facebook, WhatsApp, and Viber. A few illustrative comments about the leaflets can be found below:

“It has all the information required, very clear and with good pictures”

(Community member, Indian, Sussex)

“We've been struggling to find official NHS or government translations in Nepalese, so this is really helpful as we have a significant Nepali population locally”

(Staff, NHS Northeast Hampshire and Farnham)

“Leaflet is clear and comprehensive; I cannot wait to share among our communities and circles”

(Community Lead, Nepalese, Kent)

“Thank you so much for sharing, these leaflets are fabulous, we have shared then with our ethnic minority staff network”

(Staff, NHS Trust, Surrey)

“This is excellent work which deserves lots of appreciation.”

(Staff, NHS Trust Surrey)

## 4 Discussion

This study is, to the best of our knowledge, the first to coproduce COVID‐19 related health education resources for older adults from Indian and Nepalese communities. The first phase focused on a qualitative analysis to identify specific information needs amongst older adults from these minoritised groups. Our findings demonstrated varying levels of knowledge among participants regarding COVID‐19, with an overall acknowledgement of the need for greater awareness. Multi‐generational living was widespread and support from community members during the pandemic was highly valued. Although levels of satisfaction on the quality of care extended to them differed between individuals, desire for the integration of cultural understanding in healthcare, including the availability of resources in their languages and dissemination of health information at culturally relevant places, were consistently highlighted by the participants. The second phase of the study encompassed coproduction workshops with Indian and Nepalese communities where results from qualitative analysis to inform the development of resources were discussed. The consensus was that digital and print leaflets, containing information on COVID‐19 symptoms, transmission, protective strategies, self‐isolation, testing, and management would be most useful. The third phase of study involved resource dissemination. Team members distributed the leaflets both through post and digitally to community members, healthcare professionals, public health professionals, and researchers. Moreover, the coproduced materials were circulated by Nepalese media outlets and via social media by members of the community.

The importance of coproduction strategies in identifying gaps in care and recognising key needs of minoritsed and vulnerable communities has also been demonstrated in existing COVID‐19 related literature. A study involving minoritised ethnic communities and highly deprived communities from Middlesbrough revealed that residents attributed a great deal of importance to autonomy, community, mental wellbeing, framing of health communication, and fairness within society, while expressing a lack of trust in the official communication regarding the COVID‐19 pandemic [37]. This in turn emphasised the need to build social capital, prioritise mental health services, and frame health messaging to sound less authoritative when developing strategies to recover from the pandemic [37]. Similarly, a coproduction project found vulnerable youth to be particularly bothered by isolation during lockdown and desired better social and mental health support [38]. Moreover, African‐Caribbean, Somali, and South Asian individuals wanted COVID‐related health information to be simple, accurate, and culturally tailored with the responsibility of dissemination being handed to trusted members of the community [39]. The need for incorporating explanations regarding the disproportionate impact on minoritised ethnic communities in health communication was highlighted by these groups [39].

Co‐designed approaches have been also used to explore specific health needs among minoritised ethnic communities beyond the context of COVID‐19. A previous study focusing on mental health needs of minoritised ethnic communities suggested that providers need to acknowledge cultural and historical traumas and reach out directly to minoritised ethnic communities in appropriate contexts, using culturally tailored strategies [40]. A recent study emphasised the value of coproduction approaches in advance care planning for older adults from South Asian backgrounds [41]. The study found that older adults wanted to learn specifically about effective ways to engage in end‐of‐life conversations with their children and expressed that sessions equipping them with these skills should be delivered in‐person, in their preferred languages, in a partly didactic and partly discussion‐based format with flexibility in terms of choosing which parts of the sessions they would like to attend [41]. Similar strategies have been employed to explore the experience of using and accessing medications in marginalised and underserved communities [42]. This has led to the recognition of pervasive issues such as the lack of understanding of therapeutic effects, side effects of drugs and issues of self‐adjustment of dosage, ceasing medication altogether, stigma around attending health services and pharmacies, buying medicines from other patients, and communication challenges for non‐English speakers [42].

Coproduction helps identify the specific needs of a particular group, enabling the development of interventions and services that have greater relevance and are well received and likely to be used by the target population. We found that older adults from Indian and Nepalese communities desired culturally appropriate health resources, in turn guiding our production of culturally sensitive COVID‐19 information leaflets which were well‐received by these groups. This aligns with work by Hu et al. (2016) who reported that a culturally tailored education programme led to greater improvement in diabetes knowledge among Hispanic patients compared to a non‐culturally sensitive intervention [43]. Similarly, heightened knowledge was also noted in a group of Bangladeshi immigrants receiving culturally appropriate interventions for diabetes compared to those enroled in usual care [44]. Additionally, using a culturally relevant approach to nutrition education was found to increase health literacy, improve awareness regarding dietary fats, and enhance understanding of methods to reduce fat intake in Latinas more than interventions that did not consider their cultural needs [45]. Furthermore, Filipino individuals in the US showed higher levels of awareness about mental health disparities and increased risk of engaging in illegal drug use and unsafe sexual activity among adolescents from their communities post‐completion of a culturally tailored intervention compared to a non‐culturally relevant control [46].

Interventions grounded in cultural sensitivity have been repeatedly associated with positive health outcomes. An educational programme on type II diabetes that was responsive to the cultural needs of Hispanic communities promoted a sense of responsibility among participating individuals to commit to a healthier lifestyle and achieve better glycemic control [47]. Culturally appropriate interventions also effectively motivated African American women and South Asian men to increase their physical activity [48, 49]. Moreover, participation in a culturally tailored support group was associated with alleviation of physical and psychological symptoms and enhanced quality of life in Asian American breast cancer survivors [50]. In another study, substantially larger improvements in wellbeing, particularly depressive symptoms, were observed for culturally sensitive psychological therapy targeting minoritised ethnic communities and older adults, in comparison with usual care [51].

This study highlights the significance of increasing cultural sensitivity within healthcare has important implications for designing health‐related interventions tailored to older adults from minoritised ethnic communities. Our findings suggest that health information should be communicated in plain language with resources being made accessible in multiple formats and languages for optimal uptake and effectiveness. Interventions should incorporate elements of cultural relevance, such as multigenerational living and family structure, and recognise the benefits of these practices while addressing the unique challenges that they might pose. Time and effort should be invested by healthcare and public health professionals in building rapport and trust with target populations. Furthermore, coproduction strategies for engaging members of the target community should constitute an integral part of future resource development, from conception to dissemination.

### 4.1 Strengths and Limitations

Our study has several strengths. Firstly, the project pioneered coproduction of culturally sensitive and age‐appropriate resources for COVID‐19 health education targeting South Asian communities in the UK. Secondly, the researchers worked in partnerships with local minoritised communities through key community organisations, establishing a channel of communication with these groups and providing key lessons that contributed to more effective and tailored messaging for both communities. In coproduction process, researchers, practitioners and the public work collaboratively, flattening power hierarchy, sharing responsibility, and generating knowledge, throughout the project duration [52]. However, if the potential power asymmetry is not addressed properly, coproduction process may reproduce the existing inequalities [53]. To ensure that the older adults felt confident at expressing themselves, sufficient build up time was allowed before the start of the coproduction process through informal chats, meetings and telephone conversations. Researchers encouraged active participation to ensure that all voices of the older adults and family members were amplified and have equal participation and representation throughout the process. The opportunity to hold the meetings, interviews and workshops in Nepalese and Hindi language were provided. One of the co‐authors (NKM) is a member of the Nepalese community residing in Kent and is closely connected with the Nepalese Gurkha communities. He was trained to conduct qualitative interviews, conducted the interviews in Nepalese and contributed as a coauthor to this paper as well as to the earlier qualitative study [18]. Community members were compensated with supermarket vouchers acknowledging their time for the coproduction process.

The community‐based participatory approaches used in this study could serve as a model of coproduction and consultation to shape policy and practice for the common good of minoritised and marginalised communities more broadly. Key aspects including the principles of collaboration and equal partnerships with communities; incorporation of cultural contexts and lifestyle factors of the communities in the intervention design and allocation of sufficient time to build mutual trust could be adapted to other underserved communities. Moreover, we believe that the process, findings, and reflections from this study could be useful beyond the COVID‐19 pandemic, particularly for other seasonal outbreaks of infections (e.g., Norovirus and Influenza). Although we did not measure the full impact of the study, we believe that the leaflets may have increased knowledge within the Nepalese and Indian communities and encouraged appropriate health behaviours in the context of COVID‐19.

This study has some limitations. The literature review and websites review of the organisations conducted in phase 1 was a quick and informal to advise resource coproduction and did not include a formal systematic and comprehensive review and mapping of the literature. A systematic approach to identify and summarise the literature would have strengthened the evidence base of the coproduced resources. However, public health experts (PP, JC, KK) in the study team ensured that the messages formulated were evidence‐based and covered the changing contexts of the COVID‐19 situation. The study site was purposively selected and covered only one region in England (the Southeast), limiting the generalisability of findings to other regions with other minoritised ethnic communities or resource availability. Additionally, this study was focused on Indian and Nepalese communities and did not include other high‐risk groups (e.g., Black African), which limits the broader applicability of the findings. The leaflets were coproduced as a rapid response to the COVID‐19 situation, and before any vaccination programme had been established. The leaflets' content was based on the scientific literature at the time, government regulations, and the communities' priorities. Although some information such as using mask, physical distancing and ventilation is relevant to date, understanding of COVID‐19 and its transmission (e.g. surface transmission vs aerosol), clinical symptoms and severity (e.g., long COVID), and preventive measures (e.g., vaccines) have developed significantly since the coproduction of these leaflets, which may limit the relevance of the leaflets in the present day contexts. We acknowledge the importance of updating the information in line with evolving scientific evidence, however, due to various constraints, we were not able to update the leaflets beyond the study duration. Future research could benefit from strengthening collaborations with external partners such as local authorities, voluntary sectors, healthcare organisations etc., to maintain up‐todate resources and to ensure the sustainability of the information resources.

It was also challenging to find a meeting time for the second coproduction workshop among Nepalese community members and feedback on the process was sought using informal processes. Due to time and resources constraints, it was not possible to coproduce resources in additional formats (e.g., video messages in TV and radio etc.) as suggested by the coproduction groups. However, it was encouraging to see that one of the Nepalese communities in Scotland took the initiative to use the leaflets to develop a video resource particularly targeting older adults from Nepalese communities who are illiterate. The video can be found on the project website Co‐REM ‐ BSMS. Also, the digital copies of the leaflets were shared through emails, WhatsApp and social media. However, we did not maintain detailed records of this dissemination and could not ascertain the full reach of these resources.

Coproduction in resource limited contexts needs a careful approach, as negotiations for trade‐offs and prioritisation could impact the comprehensiveness and inclusivity of the resources. Also, this may impact the trust and relationship between the community members and researchers and the dissemination and uptake of the health information [54, 55]. However, we were not able to conduct any evaluation (again due to resource constraints) to assess these aspects and the impact of the leaflets in the communities. We acknowledge that the informal feedback and anecdotal quotes may not accurately reflect the leaflets' impact on the invovled communities. To overcome these challenges, future research should allocate sufficient resources for coproduction activities [56]. Despite these limitations, we believe that the coproduction process and the outcomes from this study have the potential to be adapted to other settings, and it would be valuable to explore what aspects of the coproduction process are transferrable and what aspects are rooted in specific cultural contexts.

## 5 Conclusion

This study provides a novel approach to coproducing culturally and age‐appropriate COVID‐19 resources to raise awareness about public health measures and potential health risks of COVID‐19 among older adults from minoritised ethnic communities in the UK. The leaflets coproduced in the study consist of information in Nepalese and Hindi covering various aspects of COVID‐19, including symptoms, protective and preventative measures, treatment, testing, and how to contact healthcare services. The leaflets were developed based on scientific evidence regarding COVID‐19 and incorporated insights from minoritised community members and healthcare professionals. By adopting an inclusive and participatory approach, the project successfully ensured that minoritised ethnic communities have accessible, acceptable, and relevant resources about the disease. The existing health inequalities related to COVID‐19, and broader determinants of health highlight the need to review how public health policies for minoritised populations are formulated and how information services are delivered in the UK. Since marginalised groups often have different needs compared to those who actively engage with services, the participatory process, findings and reflections from this study has the potential to inform future public health programmes and policies targeting other minoritised ethnic communities and marginalised groups.

## Author Contributions

P.P. and J.C. conceived the study. P.P., J.C., D.I.K., J.A., L.H., K.K., N.K.M., and R.S. obtained funding for the study. S.M.H., N.K.M., and E.S. collected the data. P.P. and A.W. prepared the final draft with input from N.K.M., J.C., D.I.K., J.A., K.K., A.S., S.G., K.C., and R.S. All authors reviewed and edited the manuscript and approved the final version. P.P. is the guarantor of this study.

## Conflicts of Interest

The authors declare no conflicts of interest.

## Data Availability

All data relevant to the study are included in the article.
